# Prevalence, diversity and transferability of the Tn*916*-Tn*1545* family ICE in oral streptococci

**DOI:** 10.1080/20002297.2021.1896874

**Published:** 2021-03-15

**Authors:** Tracy Munthali Lunde, Erik Hjerde, Mohammed Al-Haroni

**Affiliations:** a Department of Clinical Dentistry, Faculty of Health Sciences, UiT the Arctic University of Norway, Tromsø;; b ^b^Center for Bioinformatics, Faculty of Science and Technology, UiT the Arctic University of Norway, Tromsø, Norway

**Keywords:** Integrative Conjugative Elements (ICEs), oral streptococci, mobile genetic elements, antibiotic resistance, conjugation, tetracycline resistance, ddPCR, Tn*6815*, Tn*6816*

## Abstract

**Background**: The Tn*916*-Tn*1545* family of Integrative Conjugative Elements (ICE) are mobile genetic elements (MGEs) that play a role in the spread of antibiotic resistance genes. The Tn*916* harbors the tetracycline resistance gene *tet*(M) and it has been reported in various bacterial species. The increase in the levels of tetracycline resistance among oral streptococci is of great concern primarily due to the abundance of these species in the oral cavity and their ability to act as reservoirs for antibiotic resistance genes.**Methods**: In the current study, we screened 100 Norwegian clinical oral streptococcal isolates for the presence and diversity of the Tn*916*-Tn*1545* family. In addition, we investigated the transferability the elements, and the associated transfer frequencies.**Results**: We observed that 21 isolates harboured the Tn*916*-Tn*1545* family and that two of these elements were the novel Tn*6815* and Tn*6816*. The most prevalent member of the Tn*916* -Tn*1545* family observed in the Norwegian clinical oral streptococcal isolates was the wild type Tn*916*.**Conclusion**: The detection of other members of this family of ICE and varying transfer frequencies suggests high versatility of the Tn*916* element in oral streptococci in Norway.

## Introduction

The oral cavity is the second highest bacterial populated area of the human body [1]. With more than 110 approved species, streptococci are the most abundant species in the oral microbial communities [2]. In general, oral streptococci are classified as commensal bacteria and constitute part of the oral microbiota of virtually all humans. Some strains, however, have been shown to have pathogenic abilities, causing invasive infections such as infective endocarditis, septicemia, and pneumonia in neutropenic individuals [3,4]. In addition to the possible invasive pathogenicity, oral streptococci are gathering significance as they have been implicated in being reservoirs of antibiotic resistance genes [5–9], and carry substantial resistance genes as part of the ‘oral resistome’ [10]. Sequence analysis of evolutionary important genes in oral streptococci suggests that the presence of antibiotic resistance genes in these species are due, at least in part, to Horizontal Gene Transfer (HGT) [11]. One of the mechanisms of HGT involves the transfer of mobile genetic elements (MGEs) such as Integrative Conjugative Elements (ICE).

ICE constitute a very large and diverse group of MGEs of which the Tn*916*-Tn*1545* family is one of the largest members. The members of this family are by definition, self-transferable genetic elements that can exist as circular intermediates or as part of a chromosome [12–14]. Although very diverse, the elements in the Tn*916*-Tn*1545* family have Tn*916* in the backbone structure with the similar conjugative transfer, transcriptional regulation and recombination modules. Whereas Tn*916* has the *tet*(M) gene in the accessory function module, other antibiotic genes have been observed in other members of this family such as the presence of *tet*(S) in Tn*6000* [15,16] and Tn*916S* [16,17]; *erm*(B) in Tn*3872* [18]; *tet*(M) and *mef*(E) in Tn*2009* [19]; and *tet*(M), *erm*(B) and *mef*(E) in Tn*2017* [20]. Tn*916*, which was first isolated from *Enterococcus faecalis* DS16 [21], is a well-documented prototype of the Tn*916*-Tn*1545* family. It is the smallest member of the Tn*916*-Tn*1545* family and has been found in 35 different bacterial genera [22,23]. The wide spread of Tn*916*, is in part, attributed to the presence of integrase and excision genes, which enable the ICE to excise (cut) itself from one location and integrate itself in another location. The transcription regulation module of Tn*916* tightly regulates the excisionase and integrase, which are located at the 3´end of the element. This module consists of orf5, orf6, orf7, orf8, orf9, orf10 and orf12, and the importance of this module is reflected by being highly conserved [24]. It has been proposed that transcription starts from a promoter that is upstream of orf12 and runs through orf12, *tet*(M) and the downstream Open Reading Frames (ORFs) [25]. The transcription of these ORFs not only leads to the self-mobilization of the element but also increases the levels of *tet*(M), which enables the bacteria to survive in the presence of tetracyclines.

Tetracyclines, like all other antibiotics in Norway, are regulated and controlled by prescription. In 2018, tetracyclines were the second most prescribed group of antibiotics in Norway, accounting for 26% of all defined daily doses for systemic antibacterial agents [26]. Although the use of antibiotics continues to decrease in Norway, cases of antibiotic resistant bacterial isolates continue to appear [26]. This increase, coupled with the higher number of antibiotic resistance related infections highlight the need to understand the underlying factors that promote the spread and stability of tetracycline in bacterial populations.

Recent approaches to study the oral microbiome have re-enforced the abundance of *Streptococcus* ssp. in the oral cavity and the high prevalence of antibiotic resistance genes. A study by Almeida et al. 2020 [27] found that 72% of the samples isolated from the oral microbiota had at least one antibiotic resistance gene, whilst a study by Christensen and Sørensen found that more than 90% of the saliva samples analyzed carried two antibiotic resistance genes [28]. In another study, analysis of 342 clinical oral streptococci found that 44% were resistant to tetracycline, whereas 23.1% were resistant to a combination of erythromycin, tetracycline and ofloxacin [29].

The growing number of studies investigating the prevalence of resistance genes in oral streptococci illustrate the role these species play in the spread of antibiotic resistance genes. There are, however, few studies that have investigated the diversity and transferability of the Tn*916*-Tn*1545* family. In this study, we investigated the presence of the *tet*(M) carrying ICE belonging to the Tn*916*-Tn*1545* family in antibiotic resistant oral streptococci collected in Norway. We analyzed the diversity and transferability of these ICEs to determine the role that the Tn*916*-Tn*1545* family plays in the spread of these resistance determinants.

## Material and methods

### Bacterial strains

The bacterial strains used in this study were part of the oral streptococci collection strains obtained from Norwegian hospitals and submitted to the National Competence Service for the Identification of Antibiotic Resistance (K-RES). The 100 clinical strains used in this study consisted of *Streptococcus mitis* (n = 42), *Streptococcus oralis* (n = 22), *Streptococcus sanguinis* (n = 7), *Streptococcus salivarius* (n = 7), *Streptococcus anginosus* (n = 7), *Streptococcus gordonii* (n = 2), *Streptococcus constellatus* (n = 2), *Streptococcus intermedius* (n = 1), *Streptococcus mutans* (n = 1) and nine unclassified streptococcal species. In addition, the type strains *S. oralis* ATCC 35037, *S. miti*s ATCC 49456, *S. sanguinis* ATCC 10556, *S. gordonii* ATCC 10558, *S. pneumoniae* ATCC 49616, *Bacillus subtilis* BS34A (NZ_LN680001.1) and *B. subtilis* BS49 (NZ_LN649259.1) were included in this study as experimental controls.

### Construction of kanamycin resistant oral streptococcal strains

The type strains *S. oralis* ATCC 35037, *S. miti*s ATCC 49456, *S. sanguinis* ATCC 10556 and *S. gordonii* ATCC 10558 were subjected to mutagenesis using the EZ-Tn*5* < *kan-2*> transposons kit (Lucigen, Middleton, WI) according to the manufacturer’s instruction. In brief, the competent oral streptococcal cells were prepared as described by Smith et al. [30]. The EZ-Tn*5* < *kan-2*> transposon was introduced into the competent cells by electroporation using the MicroPulser Electroporator (Bio-Rad, Pleasanton, CA, USA) according to manufacturer´s instructions. The kanamycin resistant isolates *S. oralis* SOK10, *S. mitis* SMK7, *S. sanguinis* Sg10, *S. mutans* M8 and *S. gordonii* G3 were used as recipients in the Tn*916*- Tn*1545* family transferability assay.

### Assessment of Minimal Inhibitory Concentrations (MIC) of Tn916 carrying isolates

The MIC to tetracycline, erythromycin, penicillin, gentamicin, and clindamycin of the clinical oral streptococcal isolates were determined using the E-test according to the manufacturer’s instructions (BioMérieux SA, Marcy l’Etoile, France). In short, a single colony was used to prepare a fresh overnight culture in BHI broth. Bacterial cultures with a turbidity of 0.5 McFarland were inoculated onto the Mueller-Hinton-F agar supplemented with 5% horse blood, prior to the addition of the respective antibiotic strips. The agar plates were incubated in an aerobic atmosphere at 37°C for 24 hours. The isolate *S. pneumoniae* ATCC 49616 were used as a control strain. The results were interpreted according to the standards set by the European Committee for Antimicrobial Susceptibility Testing (EUCAST) (www.eucast.org) and the Clinical and Laboratory Standards Institute (CLSI) (https://clsi.org/).

### Characterization of the Tn916-Tn1545 family found in oral streptococci

The genomic DNA used in the conventional Polymerase Chain Reaction (PCR) and ddPCR was extracted using the QIAcube automated system (Qiagen, Hilden, Germany) according to the manufacturer’s instructions. To investigate the presence of the Tn*916*-Tn*1545* family in oral streptococcal strains, PCR was performed as previously described [31–33]. The presence of the *tet* (M) gene and the integrase and excisionase regions (*Int* and *Xis* genes) were determined using the primers listed in Table 1. The PCR reaction was performed in a volume of 25 µl which contained 12.5 µl DreamTaq 2X (Thermofisher Scientific), 0.5 µM of each specific primer, 8 µl of water and 2.5 µl DNA template. Subsequently, the samples that tested positive for both these targets were subjected to Long PCR using Long A primers (amplify the region between 38 bp and 9,884 bp of Tn*916*) and Long B primers (amplify position 9,824 bp to 17,947 bp of Tn*916*). These primers (listed in Table 1) were designed to detect and amplify two large fragments in Tn*916*, Tn*6002*, Tn*6003*, Tn*1545*, Tn*3878*, Tn*2009*, Tn*2010*, Tn*2017*, Tn*6084* and Tn*6079* [34]. In brief, the long PCRs were performed using Platinum SuperFI (Thermofisher Scientific) in a total volume of 25 µl. The PCR reaction mix contained 12.5 µl Platinum SuperFI mix, 5 µl Enhancer, 1 µl of each primer (10 pmol/µl), 3.5 µl water and 2 µl DNA template. The PCR reactions were run according to the manufacturer’s instructions. The two long PCR amplicons were subjected to restriction fragment length polymorphism (RFLP) by enzymatic digestion with *HincII* as previously described by Ciric et al. 2012 [34] .

**Table 1.** Details of primers and probes used

Target	Forward primer	Reverse primer	Probe sequence and label (chlorophore)	Amplicon size	Annealing temperature	Reference
*tet*(M)	GTR AYG AAC TTT ACC GAA TC	ATC GYA GAA GCG GRT CAC	N/A	633bp	55°C	[31]
*Int* and *Xis* genes	CGC CAAAGG GTC TTG TAT ATG	GCT GTA GGT TTT ATC AGC TTT TGC	N/A	650bp	58°C	[50]
Long A (position 38–9,884 bp)	GGA CTT ATC CAC TTT ATC AAG G	AAA CAG AAG CAG TGA GAA GA	N/A	9806bp	58°C	[51,52]
Long B (Position 9,824–17,947 bp)	GAA AAC TTT AGT GAT TGG TGG	CTG TAG GAA GAT ACT TCA CG	N/A	8123bp	58°C	[53]
Circular intemidate (conventional PCR)	CGT GAA GTA TCT TCC TAC A	AC CTT GAT AAA GTG TGA TAA	N/A	166bp	56°C	[54]
Circular intemidate (ddPCR)	CGT GAA GTA TCT TCC TAC A	GAC CTT GAT AAA GTG TGA TAA	AAT ACT CGA AAG CAC ATA GAA TAA GGC FAM/HEX	167bp	56°C	[41]
*Int and Xis* (ddPCR)	ATA CTC CCA TAC AGT CAA TAG TCC	AGT TCC ACC CCT GCA TGG	CCG TCG CAGGCA ATG AGT ATG GCT FAM	88bp	56°C	[41]

### Next generation sequencing of the Tn916-Tn1545 family found in oral streptococci

The extracted streptococcal DNA for the NGS was obtained using the modified Marmur extraction procedure as described by Salva-Serra et al. [35]. Based in their RFLP pattern, we selected six oral streptococcal isolates for NGS using the Illumina Nextseq 550 platform and the Pacific Biosciences Sequel instrument (PacBio) applying Sequel Polymerase v3.0, SMRT cells v3 LR and Sequencing chemistry v3.0. The Illumina Nextseq 550 was conducted at the Genomics Support Center, Tromso whereas as the PacBio sequencing was performed at the NorSeq (Oslo). Hybrid assemblies were generated using Canu [36]. The assemblies/contigs were re-ordered using Abacas 1.3.1 [37] with Tn*916* (U09422.1) as a guiding reference. The protein coding regions in the assemblies were predicted using PROKKA version 1.12 [38] and the similarities and differences between the assembled elements and the Tn*916*-Tn*1545* family were assessed using Easy fig [39]. BLAST was used to align the assembled elements to the wild type Tn*916*.

### Circularization ratio of the Tn916-Tn1545 family in oral streptococci

The circularization ratio (CR) of the Tn*916*-Tn*1545* family was determined by investigating the presence of the circular intermediate (CI) in the selected isolates. The presence of CI was determined by conventional PCR as previously described [40]. The primers used in this PCR are designed to produce an amplicon only when the element has been excised from the chromosome and the left and right junctions of the element join to form the CI. The CR was determined by ddPCR as previously described by Lunde et al. [41] and expressed as a ratio of CI to the number of the Tn*916*-Tn*1545* family elements that were present in a bacterial population.

### Transferability and conjugation frequency of the Tn916-Tn1545 family in oral streptococci

The *in vivo* transferability and conjugation frequency of the Tn*916*-Tn*1545* family was determined by filter mating experiments as previously described by Roberts et al. [42]. In brief, the donor and recipient isolates were cultivated on BHI agar plates with selection (according to the species selective markers, as listed in Table 2). The overnight cultures were prepared from single colonies and incubated at 37°C for 20 hours prior to mixing the donor and the recipient species in a 1:1 ratio. The mixed cultures were centrifuged at 1,000 g for 5 minutes and the supernatant was discarded. The cells were suspended in 1 ml of BHI broth and then 100 µl of the cell suspension was plated on a cellulose nitrate filter (0.45 µM, Sartorius Stedim Biotec; Germany). The conjugation experiments were conducted in three biological replicates and the generated transconjugants were screened phenotypically (on selective agar) and genotypically (by PCR) to verify the presence of the Tn*916*-Tn*1545* family elements. The conjugation frequency of the Tn*916*-Tn*1545* like elements was accessed both inter- and intra-species.

**Table 2.** Bacterial strains used in the inter- and intra-species transfer of the Tn*916*-Tn*1545* family to clinical and reference oral streptococci

Strain	Background and relevant phenotype	Reference
*Bacillus subtilis* BS49	Laboratory strain::Tn*916*, Tet^R^	[55]
*S. sanguinis* Ssg41	Clinical isolate:: Tn*916*, Tet^R^	[41]
*S. oralis* SO52	Clinical isolate:: Tn*916*, Tet^R^	[41]
*S. mitis* SM28	Clinical isolate:: Tn*6815*, Tet^R^	[41]
*S. oralis* SO32	Clinical isolate, Genta^R^	This study
*S. gordonii G3*	ATCC 10558::Tn*5*, Kan^R^	This study
*S. mutans M8*	ATCC 25175::Tn*5*, Kan^R^	This study
*S. mitis* SMK7	ATCC 49456::Tn*5*, Kan^R^	This study
*S. oralis* SOK10	ATCC 35,037::Tn*5*, Kan^R^	This study
*S. sanguinis* Sg10	ATCC 10556::Tn*5*, Kan^R^	This study

## Results

### Distribution of the tet(M) gene and Int and Xis genes in oral streptococcal isolates

**Figure 1.**
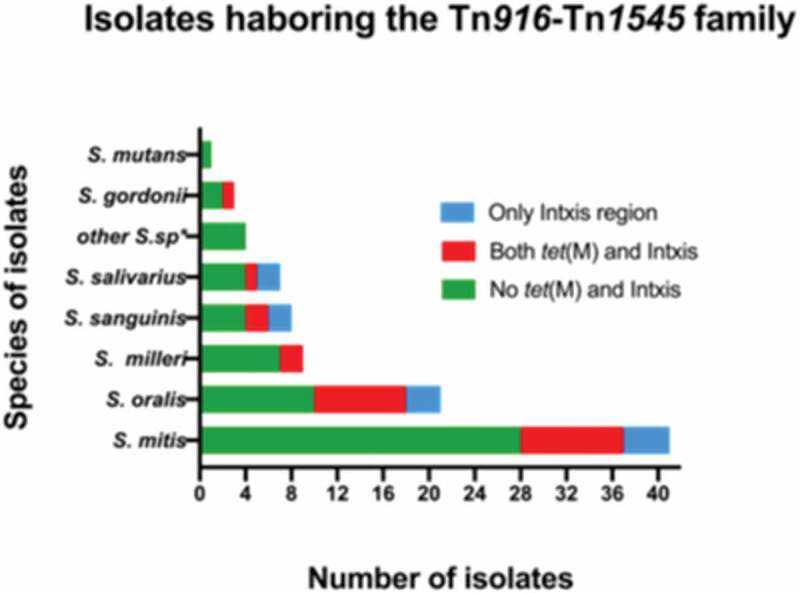
A graph illustrating the species distribution of the oral streptococci isolates included in this study. The isolates that tested PCR negative for the presence of *tet*(M), *Int* and *Xis* genes are shown in green whereas isolates that were PCR positive for *tet*(M), *Int* and *Xis* genes are illustrated in red. The blue bars indicate isolates that only tested positive for *Int* and *Xis*. S.sp*; other streptococcal species including *S. anginosus, S. constellatus* and *S. intermediate* and member of the *S. milleri* group

The 100 oral streptococcal isolates used in this study were screened for the presence of the Tn*916*-Tn*1545* family using primers that detect the *tet*(M) gene and the integrase/excisionase regions (*Int* and *Xis* genes) of these elements. A total of 24 isolates harbored *tet*(M) whereas 38 isolates carried the integrase/excisionase. The most dominant species in this study was *S. mitis* (n = 42) and the prevalence of *tet*(M) in these isolates was 26.2% (n = 11). *S. oralis* and *S. sanguinis* had a *tet* (M) prevalence of 41% (9/22) and 28.6% (2/7), respectively. Of the seven *S. salivarius*, seven S. *anginosus* and three *S. gordonii* isolates that were tested, only one isolate of each species had *tet*(M). In *S. constellatus*, only one of the two studied isolates were found to harbor the tetracycline resistance gene. The *tet*(M) gene was not detected in the tested *S. intermedius* and *S. mutans* isolates. In total, 21% (21/100) tested positive for both the *tet*(M) gene and the integrase/excisionase region and these were considered to harbor Tn*916*-Tn*1545* like elements. The species distribution of both *tet*(M) and the integrase/excisionase regions is illustrated in Figure 1.

### Assessment of minimal inhibitory concentrations of Tn916 carrying isolates

The antibiotic resistance profile of the 21 isolates harboring the Tn*916*-Tn*1545* family were assessed by determining the MIC to tetracycline, erythromycin, penicillin, gentamicin and clindamycin, and the result are shown in Table 3. The MIC to tetracycline ranged from 0.190 to 96 μg/ml. All isolates that carried the *tet*(M) gene were phenotypically resistant to tetracycline with the exception of isolate *S. oralis* SO04 where the *tet*(M) gene was silent and the detected MICs were below the resistance threshold (≤ 2 μg/ml) as determined by EUCAST. The MIC values to erythromycin ranged from 0.023 to >256 μg/ml and of the 21 isolates, five were classified as resistant to erythromycin with MIC values ranging from 3 to > 256 μg/ml. The MIC to penicillin, gentamycin and clindamycin where found to be 0.016 to 1.5 g/ml, 0.16 to 32 μg/ml and clindamycin 0.094 to > 256 μg/ml, respectively. Although most strains were resistant to more than two antibiotics, only 16% (n = 4) of the tested isolates were resistant to four of the five tested antibiotics. The isolate *S. mitis* SM28 was the only isolate found to be phenotypically resistant to penicillin with a MIC of 6 μg/ml.

**Table 3.** Antibiotic resistance profile of oral streptococcal isolates harboring the Tn*916*-Tn*1545* family collected in Norway

Isolate	Tetracycline μg/ml	Erythromycin μg/ml	Penicillin μg/ml	Gentamycin μg/ml	Clindamycin μg/ml
*S. sanguinis* SS33	24 (R)	0.047 (S)	0.190 (S)	6 (IE)	0.125 (S)
*S. sanguinis* SS41	32 (R)	0.016 (S)	0.047 (S)	1.5 (IE)	0.160 (S)
*S. oralis* SO1	32 (R)	>256 (R)	0.016 (S)	16 (IE)	>256 (R)
*S. oralis* SO4	0.190 (S)	0.125 (S)	0.023 (S)	12 (IE)	0.190 (S)
*S. oralis* SO30	24 (R)	0.047 (S)	0.094 (S)	8.00 (IE)	0.094 (S)
*S. oralis* SO44	24 (R)	3 (R)	1 (I)	24 (IE)	0.094 (S)
*S. oralis* SO47	32 (R)	0.047 (S)	0.125 (S)	32 (IE)	0.094 (S)
*S. oralis* SO52	32 (R)	0.094 (S)	0.064 (S)	24 (IE)	0.125 (S)
*S. oralis* SO62	2 (S)	6 (R)	0.047 (S)	24 (IE)	0.75 (R)
*S. oralis* SO67	64 (R)	0.047 (S)	0.125 (S)	12 (IE)	0.094 (S)
*S. oralis* SO69	96 (R)	6 (R)	1.500 (I)	32 (IE)	0.125 (S)
*S. oralis* SO90	2 (S)	0.125(S)	0.064 (S)	12 (IE)	0.32 (S)
*S. mitis* SM2	48(R)	>256 (R)	0.094 (S)	0.16 (IE)	>256 (R)
*S. mitis* SM28	64 (R)	>256 (R)	6 (R)	6 (IE)	>256 (R)
*S. mitis* SM29	32 (R)	0.047 (S)	0.047 (S)	1.5 (IE)	0.064 (S)
*S. mitis* SM74	2 (S)	0.064 (S)	0.380 (I)	24 (IE)	0.094 (S)
*S. mitis* SM81	48 (R)	0.032 (S)	0.190 (S)	6 (IE)	0.125 (S)
*S. gordonii* SG71	24 (R)	0.047 (S)	0.016 (S)	4 (IE)	0.064 (S)
*S. anginosus* SA46	12 (R)	0.094 (S)	0.047 (S)	12 (IE)	0.094 (S)
*S. salivarius* SSv51	96 (R)	0.064 (S)	0.023 (S)	12 (IE)	0.125 (S)
*S. constellatus* SC99	16 (R)	0.023 (S)	0.094 (S)	3 (IE)	0.047 (S)

S = Susceptible; I = Intermediate; R = Resistant; IE = Intrinsic Resistance.

### Characterization of the Tn916-Tn1545 family found in oral streptococci

The Tn*916*-Tn*1545* family elements were characterized by amplifying two fragments that covered a large portion of the 18 kb wild type Tn*916*; Long A (position 38 bp to 9,844 bp) and Long B (position 9,824 to 17,947 bp). When subjected to RFLP analysis, the large amplicons indicated that 15 out of the 21 isolates harbored the wild type Tn*916*. The RFLP pattern of isolates *S. oralis* SO62, *S. oralis* SO67, *S. mitis* SM74, *S. oralis* SO90, *S. mitis* SM28 and *S. constellatus* SC99 varied from the wild type Tn*916* (as shown in Supplementary Figure S1). This suggested that these isolates potentially harbor other members of the Tn*916*-Tn*1545*family and were thus selected for further analysis with NGS.

### Next generation sequencing of the Tn916-Tn1545 family found in oral streptococci

Analysis of the NGS resulted in the assembly of complete ICE in all of the isolates with the exception of isolate *S. oralis* SO62. The assembled elements are available under the Bioproject 660235 on NCBI. Sequence comparisons of the wild type Tn*916* with the entire length of Tn*916*-Tn*1545* like elements found high similarity in the isolates *S. mitis* SM74 and *S. oralis* SO90 of 98.8% and 98.8%, respectively. Analysis of the *S. oralis* SO67 assembled genome, showed the presence of two Tn*916* elements. The elements were completely identical and showed 99.8% sequence identity to Tn*916* with 99% query coverage (17,994/18,032). The resulting variations in the RFLP patterns compared to the wild type Tn*916* were found to be due to SNPs and *in silico* digestion of the sequenced elements confirmed the obtained RFLP pattern.

**Figure 2.**
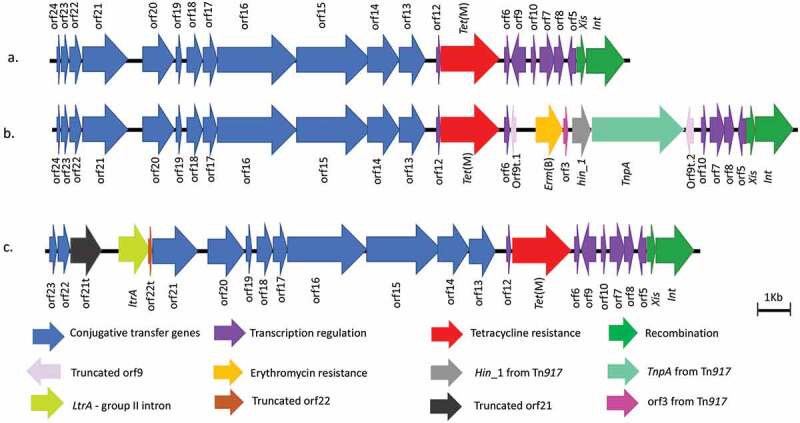
A schematic representation of the structure of Tn*916* and the Tn*916*-Tn*1545* family elements. The image in A. is Tn*916* from *E. faecalis* DS16 (accession number U09422.1); B. illustrates Tn*6815* from *S. mitis* SM28 (accession number LR828204.); and C. Tn*6816* from *S. constellatus* SC99 (accession number LR828206.1). The element Tn*6815* carries the *erm*(B) *hin_1*, orf3 and *TnpA* Transposase from Tn*917* inserted between two truncated regions of orf9 (orft.1 and orft.2). The element in *S. constellatus* SC99 has been designated Tn*6816* and it carries group II intron reverse transcriptase/maturase, truncated orf21 and orf22 and a complete copy of orf21 inserted upstream of orf20

In *S. mitis* SM28, an insertion of 5,265 bp was observed respectively as shown in Figure 2. Sequence comparison of the assembled element in S. *mitis* SM28 showed 99.3% similarity with ICESpn22664 (accession number HG799489.1) [43] with a coverage of 98%. The element was also found to harbor the *erm*(B), orf3, hin_1 and *tnpA* (a transposase) genes which are inserted within orf9 and are located downstream of *tet*(M). The Blast results of this region showed high similarity (99.89%) to Tn*917* (accession number M11180.2). This suggested that Tn*6815* is a composite element in which Tn*917* has been inserted into position 14,525 bp of Tn*916* and extends to position 19,790 bp. Downstream of position 19,790 bp, the element was identical to the 3´end of Tn*916*. This element has been given a new name, Tn*6815*, and registered in the Transposon registry [44]. The sequence of the novel Tn*6815* is available on NCBI under the accession number LR828204.1

BlastN alignment between LR828204 (Tn*6815*) and U09422 showed that Tn*916* covered 77% of Tn*6815* with two matches going from 1 to 14,524 to the left of the 5,266 bp insertion and 19,790 to 23,299 right of the insertion. The 5´end of the alignment ranged from 1 to 14,524 in both elements and was 99% identical whereas the 3´end after the insertion was 98% identical to Tn*916* and corresponded to positions 14,519 bp to 18,032 bp in Tn*916*. The Tn*6815* carried two truncated orf9 segments which have been designated orf9t.1 and orft.2. The orf9t.1 was 136 bp and lacked 218 bp of orf9 and the orf9t.2 lacked the first 131 bp of the 5´end but had the remaining 222 bp of the 3´end. The right side of the alignment started at 14,519 (which is 131 bp within orf9) and covered the rest of Tn*916*. The Tn*6815* also carried a 5 bp repeat of orf9 on either side of the 5,266 bp insertion. BlastN analysis showed that the insertion was 99% identical to Tn*917* (accession number M11180.2) and that three ranges were present in Tn*6815*. The first range started at 90 bp and went to 5,355 bp, which is the entire sequence of Tn*917*. The second range was from 92 bp to 162 bp (corresponded to 15,988 bp to 16,058 bp in Tn*6815*) and the third range was from 1,550 bp to 1,623 bp which corresponded to 14,524 bp to 14,597 bp in Tn*6815*. These last two ranges were 96% identical (68/71bp similar) indicating that there was a repeated segment with Tn*917*.

In *S. constellatus* SC99, an element harboring a 1,663 bp insertion was observed. The insertion was located 401bp before the end orf21 and corresponded to the *ltrA* gene, a group II intron reverse transcriptase/maturase and part of peptidase P60. The *ltrA* gene was followed by 91bp of the 3´end of orf22 (lacking 295 bp of orf22) and a complete copy of orf21. BlastN analysis indicated that the element had two copies of orf21and orf22. The first copy of orf21 was truncated and only 1,061 bp long. It has been designated orf21t and was located upstream of the inserted *ltrA* and downstream of the first and complete copy of orf22. The second copy of orf21 was complete and located downstream of *ltrA*. Sequence comparison of this insertion showed 99.9% similarity and 100% coverage with the *ltrA* gene found in the *Enterococcus avium* strain 352 chromosome (accession number CP034169.1). As no matches of 99% or more were obtained from sequence comparison analysis, this element has been designated a new name, Tn*6816*, and registered in the Transposon registry [44]. The sequence of the novel Tn*6816* is available on NCBI under the accession number LR828206.1

### Circularization ratio of the Tn916-Tn1545 family in oral streptococci

**Figure 3.**
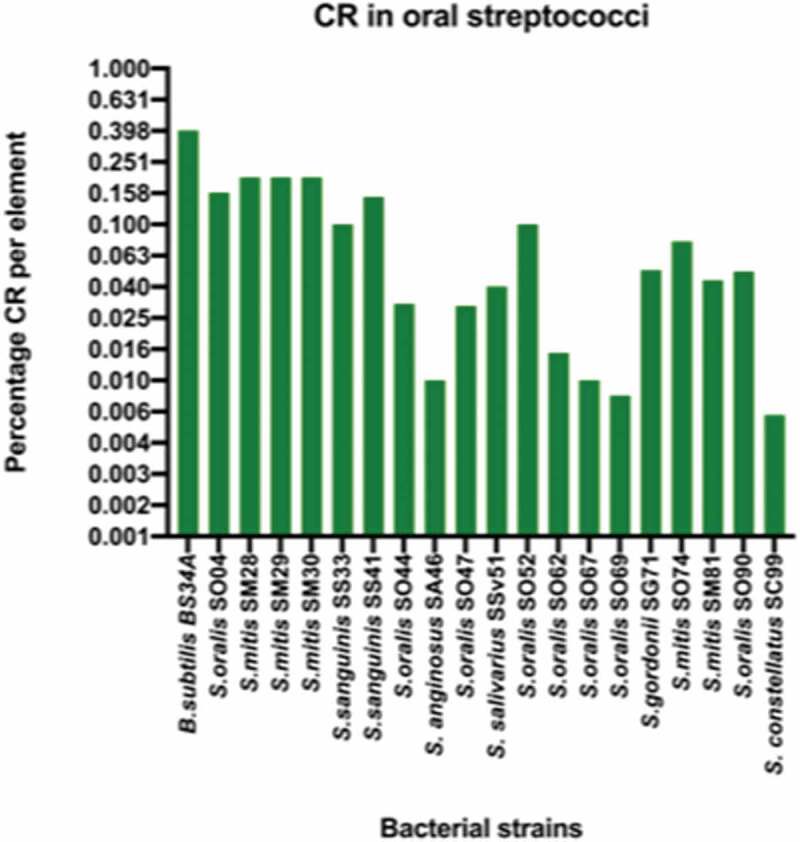
A graph illustrating the percentage of circularization rate (CR) in oral streptococci. *B. subtilis* BS34A was used as a positive control for the excision of Tn*916* and shows the highest levels of CR. The clinical oral streptococci isolates have CR that ranges from zero to 0.25 percentage CR per element

The presence of CI was first determined by conventional PCR and CR was determined by ddPCR. In the absence of selective pressure, the conventional PCR results indicated the presence of the CI molecules in all the tested isolates with the exception of *S. mitis* SM74. The quantification of the CI molecules in the bacterial population by ddPCR was found to range from 0% in two isolates to 0.2% per element as indicated in Figure 3. Interestingly, the three highest levels of CI observed in this study were from *S. mitis* isolates. This coupled with the fact that *S. mitis* is one of the most abundant streptococcus species in the oral cavity warranty the need to further investigate the evolution of Tn*916* in *S. mitis*.

### Transferability and conjugation frequency of the Tn916-Tn1545 family in oral streptococci

The *in vivo* transferability of the Tn*916*-Tn*1545* family was investigated by filter mating experiments in which both laboratory and clinical isolates were used as donor strains. The strain *B. subtilis* BS49 was included in this study as a control strain for the transfer of Tn*916* as this element is known to be transferable with a CR of 9.7% [41], and exists as two copies [45]. To evaluate the transferability of Tn*916*-Tn*1545* elements in the clinical setting, we used clinical isolates harboring the wild type Tn*916* as donor strains in the conjugation experiments. Of the 30 filter mating experiments, 28 yielded transconjugants with frequencies that are summarized in Table 4. The bacterial strain *B. subtilis* BS49 was able to transfer Tn*916* to five out of the six recipients. Interestingly, the isolate in which no transfer was detected (*S. oralis* SO32) is a clinical isolate whereas the other recipients are laboratory strains. In the successful conjugation experiments, the transfer frequency of Tn*916* from *B. subtilis* BS49 ranged from 6.0 (± 4.03) × 10^−9^ to 1.5 × 10^−1^ (±0.25). The frequencies of transfer ranged between to 4.0 (± 3.0) × 10^−7^ to 3.5 (± 6.0) × 10° per recipient when the clinical isolate *S. sanguinis* Ssg41 harboring Tn*916* was used as a donor. The transfer of Tn*916* from the clinical isolate *S. oralis* SO52 was observed to ranged from 8.4 × 10^−3^ (±0.01) to 4.7 (± 4.1) × 10^−1^. The transfer frequency of Tn*6815* ranged between 7.4(± 9.7 × 10^−6^ and 5.8 (± 3.6) × 10^−2^ whereas for Tn*6516*, the transfer frequencies ranged between below detection in *S. oralis* SO32 to 1.5 (± 2.7) × 10^−1^ in *S. sanguinis* Sg10.

**Table 4.** Transfer frequencies of the Tn*916*-Tn*1545* family in oral streptococci per recipients

Donor (element)	*S. oralis* SOK10	*S. mitis* SMK7	*S. sanguinis* Sg10	S. *gordonii* G3	S. *mutans* M8	S. *oralis* SO32
*Bacillus subtilis* BS49; Tet^R^ ::Tn*916*	4.7 (± 8.1) × 10^−6^	6.0 (± 4.03) × 10^−9^	2.9 (± 4.9) × 10^−5^	1.5 (±0.25) × 10^−1^	3.2 (± 4.1) × 10^−6^	Not detected
*S. sanguinis* Ssg41; Clinical isolate Tet^R^ :: Tn*916*	3.5 (± 6.0) × 10°	1.7 (± 1.8) × 10^−1^	1.0 (±0.09) × 10^−6^	1.0 (± 1.7) × 10^−3^	3.0 (± 4.6) × 10^−6^	4.0 (± 3.0) × 10^−7^
*S. oralis* SO52; Clinical isolate Tet^R^ :: Tn*916*	3.1 (± 2.3) × 10^−2^	2.1 (± 1.2) × 10^−2^	6.2 (±0.09) × 10^−2^	4.7 (± 4.1) × 10^−1^	6.2 (±0.09) × 10^−2^	8.4 (±0.01) × 10^−3^
*S. mitis* SM28; Clinical isolate Tet^R^ ::Tn*6815*	2.9 (± 3.1) × 10^−3^	2.5(± 3.9) × 10^−3^	7.4 (± 9.7) × 10^−6^	1.4 (± 3.5) × 10^−3^	3.3 (± 5.4) × 10^−2^	5.8 (± 3.6) × 10^−2^
*S. constellatus* SC99; Clinical isolate :: Tet^R^ Tn*6816*	2.2 (± 1.9) × 10^−3^	1.2 (± 2.1) × 10^−2^	1.5 (± 2.7) × 10^−1^	1.0 (± 1.6) × 10^−1^	1.8 (± 2.0) × 10^−3^	Not detected

Tet^R –^ Tetracycline resistant

**Figure 4.**
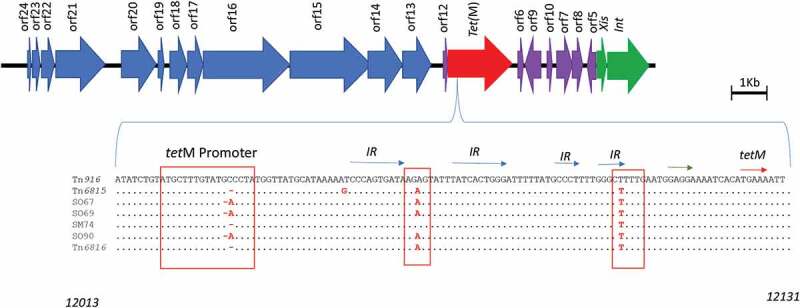
A nucleotide alignment of the promoter region in Tn*916* and six oral streptococci clinical isolates highlighting single nucleotide changes in the clinical isolates. The dots indicate nucleotides that are similar to the reference Tn*916* (KM615585), whereas the red nucleotides indicate SNPs. The red boxes indicate seemingly conserved SNPs, the blue arrows; inverted repeats (IR), the green arrow; the ribosomal protein binding site, and the red arrow; the start of the tetracycline resistance gene *tet*M. The numbers in the bottom right and left corners of the image indicate the nucleotide position in the reference *E. faecalis* DS16 (U09422.1). Tn*6815* and Tn*6816* are novel transposons that have been isolates from the clinical isolates *S. mitis* SM28 and *S. constellatus* SC99

The random selection of twenty colonies from the resulting transconjugants per filter mating confirmed the presence of both the *tet*(M) and the *Int* and *Xis* genes of Tn*916* by conventional PCR. In addition, growth of the transconjugants on BHI agar plates containing both selection makers, i.e. tetracycline and kanamycin, verified the transfer of the Tn*916*-Tn*1545* family to the recipient cells. Sequence analysis of the *tet* (M) promoter region in all the selected clinical isolates, which is known to influence the rate of transfer, showed nucleotide changes as shown in Figure 4.

## Discussion

Oral streptococci are the most abundant bacterial species in the oral cavity and are regarded as commensal bacteria. *Streptococcus* species can, however, cause serious infections if they enter the blood stream. There has been an increase in the number of antibiotic resistant oral streptococci thus the aim of this study was to determine the prevalence of the Tn*916*-Tn*1545* family in a collection of oral streptococci from Norway. The presence of *tet*(M) was detected in 24% the 100 oral streptococcal isolates. This rate of carriage is within the previously reported prevalence of 21% and 29.6% of *tet*(M) in oral streptococci [46,47]. The above-mentioned studies were from 1984 and 2017 respectively, therefore it is interesting that despite the time span, different geographic locations and changes in usage of antibiotics, the carriage of *tet*(M) appears to be similar. Of the 100 investigated isolates, the Tn*916*-Tn*1545* family was detected in 21% of the isolates with the highest prevalence per species occurring in *S. oralis* (41%). *S. mitis* was the most prevalent species in this study (41%), however only 26.2% of the isolates harbored the Tn*916*-Tn*1545* family. This raises the question of whether the elements have species preference or not, a notation that requires further study. Analysis of the diversity of Tn*916*-Tn*1545* in our study, revealed a total of three different elements with the most occurring being Tn*916.* The other two elements are new members in the Tn*916*-Tn*1545* family, i.e. Tn*6815* and Tn*6816*. Other studies have documented a diversity of four elements in a collection of 48 oral streptococci where the most frequent occurring was Tn*3872* [34].

The presence of both tetracycline and erythromycin resistance genes in Tn*6815* and *ltrA* in Tn*6816* is an indication of the role that these elements play in the elasticity of the bacterial genome and HGT. Although the diversity of these elements in our study seems low, it should be noted that based on the inclusion criteria set for further analysis for the Tn*916*-Tn*1545* family excluded the analysis of 14 oral streptococci that tested positive only for the integrase/excisionase regions and not *tet* (M). It can be speculated that including these samples in this study might have increased the diversity of the detected elements.

Based on the RFLP analysis, the most prevalent form of the Tn*916*-Tn*1545* family observed was the prototype Tn*916*, which was present in 15 isolates. Interestingly, despite carrying the same element, the 15 isolates had different MIC and lower levels of the CI when compared to the wildtype of *B. subtilis* BS34. As in many other clinical isolates, the variations in the level of resistance to tetracycline may be attributed to several factors such as the presence of other tetracycline resistance genes in the chromosome and/or the strength of the *tet* promoter [25]. Analysis of the *tet*(M) promoter regions in the sequenced isolates (Figure 4) illustrates some of the SNPs that we identified. The fact that these SNPs were observed in nearly all the analyzed clinical isolates, and since it has been hypothetized that their presence is due to beneficial evolutionary advantages requires further analysis and investigations .

Although all but one of the Tn*916* like elements found in this study harbored only one resistance gene, the antibiotic resistance profile indicated that five isolates were resistant to erythromycin and that four of the tested isolates were resistant to four of the five tested antibiotics. This may suggest the presence of other MGE or the presence of other resistance genes within the bacterial chromosomes and further highlights the role that oral streptococci play in the spread of antibiotic resistance among bacteria.

The six isolates that were identified as having *Tn916-*like elements based on the observed difference in the RFLP were further investigated by NGS. The results from the NGS showed that in three of the six isolates (isolates *S. oralis* SO67, *S. mitis* SM74 and *S. oralis* SO90), the observed differences in the RFLP are due to SNPs and/or mutations that result in the introduction of a new *HincII* digestion target site. According to the sequences of these isolates, they were more than 99% similar to the prototype Tn*916*. These observations highlighted the need to consider the suitability of the using the RFLP method as a way of distinguishing Tn*916* from Tn*916*-like elements. In the isolate *S. oralis* SO67, assembly of the genome, suggested the presence of two identical copies of Tn*916* in two different chromosomal locations. According to our knowledge, this is the first time that two identical copies of Tn*916* have been identified in two different positions in the same chromosome of an oral streptococcal clinical isolate.

*S. mitis* is one of the most predominant oral *Streptococcus* species in the oral cavity. The isolate *S. mitis* SM28 was found to harbor the novel Tn*6815* which contained the *erm*(B) gene inserted downstream to *tet*(M). The presence of *erm*(B) and *tet*(M) genes rendered *S. mitis* SM28 the only isolate found to have two resistance genes in the same element in this study. Tn*6815* harbored two truncated copies of orf9. In spite of the absence of a complete orf9, Tn*6815* was able to form CI and excise from the genome suggesting that an alternative mechanism is regulating the excision process, as has been suggested to occur in other Tn*916*-like elements [23]. The 5,265 bp insertion downstream from the *tet*(M) gene resulted in an element that was 99% identical to an ICE that has been previously isolated from *S. pneumonia* [43]. In contrast with the other previously identified elements that carry insertions downstream from the *tet*(M) gene such as Tn*2009* and Tn*3872* [19], the Tn*6815* in *S. mitis* SM28 was transferable to other oral streptococcal strains (Table 4). In addition to carrying both the *tet*(M) and the *erm*(B) genes, *S. mitis* SM28 was also shown to be resistant to penicillin, which is usually the first drug of choice for oral streptococcal infections. The ability of Tn*6815* to transfer to other species, further supported previous findings that oral streptococci play an active role in the dissemination of resistance genes by being reservoirs of antibiotic resistant genes.

The formation of the CI is the first step in the transfer of the Tn*916*-Tn*1545* elements, hence the presence of the CI and CR may indicate the functionality of the ICE to disseminate associated genes, including resistance genes. To show that the element is transferable, we conducted *in vivo* inter- and intra-species conjugation assays. Interestingly, we were able to show both inter- and intra-species transfer of Tn*916*-Tn*1545* elements with transfer frequencies ranging from 6.0 (± 4.03) × 10^−9^ to 3.5 (± 6.0) × 10° (Table 4). Conjugation frequencies of Tn*916* to *S. gordonii, S. salivarius* and *S. sanguinis* have previously been reported to range from 10^−8^ to 10^−5^ [48,49]. Although some of the observed conjugation frequencies reported in this study were comparable to those previously reported, 20/28 of the observed conjugation frequencies were considered higher than what has been previously reported [48,49]. Interestingly all the higher observed conjugation frequencies where observed when clinical isolates where used as donor suggesting that clinical isolates may be more efficient in spreading Tn*916* despite having a lower CR. In spite of the bacterial isolate *B. subtilis* BS49 having two copies of Tn*916* and having higher CR than the clinical oral streptococci in this study, it was observed that the *B. subtilis* BS49 yielded the lowest number of transconjugants compared to the studied oral streptococci. In this study, we observed varying transfer frequencies of the Tn*916*-Tn*1545* elements within the same recipients when different donors were used in the filter mating and vice versa. These findings suggested that other factors than the donor, recipient, number of elements and/or the number of CI influence the rate at which the Tn*916*-Tn*1545* family elements are transferred within and between species.

The clinical oral *Streptococcus* isolates were all found to have a low CR. Sequence analysis of the *tet*(M) promoter region in all the selected clinical isolates revealed the presence of some SNPs (Figure 4). As this region has been shown to influence the transcription and transfer of the element, the observed SNPs might be responsible for the observed low levels of CR.

The presence of the Tn*916*-Tn*1545* family in commensal oral streptococci raises concern as it highlights the role that these abundant species may play in the increasing rates of antibiotic resistance. In this study, 21% of the investigated oral streptococci have been found to harbor a diverse elements of the Tn*916*-Tn*1545* family, with the highest prevalence occurring among *S. mitis* isolates. One of these *S. mitis* isolates was found to harbor a new element, i.e. Tn*6815* that had two resistance genes, i.e *tet*(M) and *erm*(B). Tn*6815* is transferable to other species with a relatively high transfer frequency. These findings underscore the role of *S. mitis* plays in the spread of antibiotic resistance within and across bacterial species. There is a need to further investigate the factors that contribute to the stability of the Tn*916*-Tn*1545* elements in oral streptococci.

## Supplementary Material

Supplemental Material
