# Supplementary material for: Prevalence, diversity and transferability of the Tn916-Tn1545 family ICE in oral streptococci
Source: J Oral Microbiol. 2021 Mar 15;13(1):1896874. doi: 10.1080/20002297.2021.1896874 (PMC7971310; doi:10.1080/20002297.2021.1896874)
Supplement: Supplemental Material [file ZJOM_A_1896874_SM2429.docx]

Supplementary data S1: RFLP digestion of the Tn*916*-Tn*1545* family in oral streptococci

B

A

RFLP of the long A (position 38bp- 9884bp) in image A and long B (position 9824- 17947bp) in image B, of Tn*916-*Tn*1545* family in the oral streptococci isolates after digestion by HincII. The band patterns illustrate the similarities and/or difference of the element in the test strains in relation to the positive control *B. subtilis* BS49 in lane 2. In Lane 1: 1Kb plus molecular weight marker (NEB); Lane 3: *S. constellatus* SC99; Land 4: *S. mitis* SM02; Lane 5: *S. oralis* SO04; Lane 6: *S. mitis* SM28; Lane 7: *S. oralis* SO30, Lane 8: *S. sanguinis*

Ssg33; Lane 9: *S. sanguinis* Ssg41; Lane 10: *S. oralis* SO47; Lane 11: *S. oralis* SO52; Lane 12: *S. oralis* SO62; Lane 13: *S. oralis* SO67; Lane 14: *S. oralis* SO69; Lane 15: *S. gordonii* SG71; Lane 16: *S. mitis* SM74; Lane 17: *S. mitis* SM81; Lane 18: *S. oralis* SO90 and Lane 19: *S. salivarius* Ssv51.
